
JOURNAL INFORMATION
==============================
NLM Title Abbreviation: ScientificWorldJournal
Journal ID: ScientificWorldJournal
NLM Journal ID: 101131163
Journal ID: TSWJ

The Scientific World Journal

ISSN: 2356-6140
EISSN: 1537-744X
Publisher: Wiley

ARTICLE INFORMATION
==============================
PMCID: PMC6084163
PMID: 30147583
DOI: 10.1100/tsw.2001.129
Article ID: 741526
Article version: 1
Subjects: Short Report

BCL-2 AND IAP Proteins As Potential Drug Targets

Fesik Stephen W. *
Pharmaceutical Discovery Division, Abbott Laboratories, Abbott Park, IL 60064, USA
*Stephen W. Fesik: stephen.fesik@abbott.com
Publication date: 2001
Electronic publication date: 2001 Feb 9
Volume: 1
First page: 105
Last page: 105
Copyright: Copyright © 2001 Stephen W. Fesik.
Copyright year: 2001
License: This is an open access article distributed under the Creative Commons Attribution License, which permits unrestricted use, distribution, and reproduction in any medium, provided the original work is properly cited.
License URL: https://creativecommons.org/licenses/by/3.0/

==============================
Miami *Nature Biotechnology* Short Reports
*TheScientificWorld* (2001) 1(S3), 105SR
ISSN 1532-2246; DOI 10.1100/TSW.2001.129

### BCL-2 AND IAP PROTEINS AS POTENTIAL DRUG TARGETS

Stephen W. Fesik

Pharmaceutical Discovery Division, Abbott Laboratories, Abbott Park, IL 60064, USA
Stephen.Fesik@abbott.com

**INTRODUCTION**. Members of the Bcl-2 and IAP families of proteins inhibit apoptosis and are overexpressed in many cancers. Thus, small molecules that bind to these proteins and block their anti-apoptotic activity may induce cell death and be useful for the treatment of cancer. To aid in the design of these small molecules that bind to these proteins, we have determined the three-dimensional structures of Bcl-2 and IAP family members alone and when complexed to inhibitors (1-6).

**METHOD**. Three-dimensional structures of the anti-apoptotic proteins were determined using heteronuclear multi-dimensional NMR spectroscopy. NMR spectra were acquired on a Bruker 500, 600, or 800 MHz NMR spectrometer. The backbone and side chains were assigned using double and triple resonance experiments, and distance restraints were obtained from 3D NOESY experiments. Structures were calculated with a torsion angle dynamics and simulated annealing protocol using NMR-derived distance and angular restraints.

**RESULTS**. Three-dimensional structures of Bcl-xL, Bcl-2, Bcl-xL/Bak peptide, and Bcl-xL/Bad peptide complexes were obtained. The overall structures of Bcl-xL and Bcl-2 are similar and consist of two central, primarily hydrophobic α-helices surrounded by amphipathic α-helices. The Bak and Bad peptides bind in a hydrophobic groove in the anti-apoptotic proteins. NMR structures of the Bir2 and Bir3 domain of XIAP were also obtained. The structures of the XIAP Bir domains resemble classical zinc fingers and are composed of a three-stranded anti-parallel β-sheet and several α-helices.

**DISCUSSION**. The structures of these anti-apoptotic proteins reveal binding pockets where small molecules could potentially interact with these proteins. This structural information is useful for designing small molecules that bind to the Bcl-2 and IAP family members. These molecules may be useful for treating cancers.

**REFERENCES.**

1. Muchmore, S.W., Sattler, M., Liang, H., Meadows, R.P., Harlan, J.E., Yoon, H.S., Nettesheim, D., Chang, B.S., Thompson, C.B., Wang, S.L., Ng, S.C., and Fesik, S.W. (1996) Nature 381, 335-341
2. Sattler, M., Liang, H., Nettesheim, D., Meadows, R.P., Harlan, J.E., Eberstadt, M., Yoon, Y.S., Shuker, S.B., Chang, B., Minn, A.J., Thompson, C.B., and Fesik, S.W. (1997) Science 275, 983-986
3. Sun, C., Cai, M., Gunasekera, A.H., Meadows, R.P., Wang, H., Chen, J., Zhang, H., Wu, W., Xu, N., Ng. S.C., and Fesik, S.W. (1999) Nature 401, 818-822
4. Fesik, S.W. (2000) Cell 103, 273-282

5. Sun, C., Cai, M., Meadows, R.P., Xu, N., Gunasekera, A.H., Wu, J., Hermann, J., and Fesik, S.W. (2000) J. Biol. Chem. (in press)

6. Petros, A.M., Nettesheim, D.G., Wang, Y., Olejniczak, E.T., Meadows, R.P., Mack, J., Swift, K., Matayoshi, E.D., Zhang, H., Thompson, C.B., and Fesik, S.W. (2000) Protein Sci. (in press).
